# Integrating UTAUT and TTF models with personal innovativeness to examine smart home appliance adoption for sustainable consumption

**DOI:** 10.1038/s41598-026-51248-w

**Published:** 2026-05-06

**Authors:** Yao-Wu Wang, Cheng-Min Chao, Bo-siang Chen, Tzu-Hsin Chu, Wei-Sho Ho, Wei-Lun Huang

**Affiliations:** 1 Department of Electrical and Mechanical Technology, National Changhua University of Education, Bao-Shan Campus, No.2, Shi-Da Rd, Changhua City, 500208 Taiwan; 2 Department and Graduate Institute of Information Management, Yu Da University of Science and Technology, No. 168, Hsueh-fu Rd., Tanwen Village, Chaochiao Township, Miaoli County, Miaoli County, 361027 Taiwan; 3 Department of Business Administration, National Taichung University of Science and Technology, No. 129, Sec. 3, Sanmin Rd., North Dist, Taichung City, 404336 Taiwan; 4 Department of Vehicle Engineering, Nan Kai University of Technology, No. 568, Zhongzheng Rd., Caotun Township, Nantou City, 542020 Taiwan; 5 Graduate Institute of Technological and Vocational Education, National Changhua University of Education, Bao-Shan Campus, No.2, Shi-Da Rd, Changhua City, 500208 Taiwan; 6 NCUE Alumni Association, National Changhua University of Education, Jin-De Campus, No. 1, Jinde Rd, Changhua City, 500207 Taiwan; 7 Medical Affairs Office, National Taiwan University Hospital, No. 7, Zhongshan S. Rd., Zhongzheng Dist, Taipei City, 100225 Taiwan; 8 Department of Health Services Adminstration, China Medical University, No. 100, Sec. 1, Jingmao Rd., Beitun Dist, Taichung City, 406040 Taiwan; 9 Department of Health Care Management, National Taipei University of Nursing and Health Sciences, No. 365, Mingde Rd., Beitou Dist, Taipei City, 112303 Taiwan

**Keywords:** Smart home appliances, Task-Technology Fit (TTF), UTAUT, Personal innovativeness, Sustainable consumption, PLS-SEM, Technology adoption, Business and management, Business and management, Information systems and information technology, Psychology, Psychology, Science, technology and society

## Abstract

Smart home appliances are pivotal for advancing energy efficiency and environmental sustainability; however, their widespread adoption faces complex psychological and functional barriers. This study investigates the key determinants of consumers’ continuous usage intentions by integrating the Unified Theory of Acceptance and Use of Technology (UTAUT) and the Task-Technology Fit (TTF) model. To comprehensively capture consumer behavior, the theoretical framework is extended to incorporate trust, perceived source credibility, and personal innovativeness. Utilizing partial least squares structural equation modeling (PLS-SEM), data collected from 1,919 consumers in Taiwan were analyzed. The empirical results demonstrate that both task and technology characteristics significantly enhance TTF. Consequently, a higher TTF positively influences performance expectancy, effort expectancy, social influence, and facilitating conditions. Furthermore, trust and perceived source credibility serve as critical antecedents to performance expectancy, while personal innovativeness significantly strengthens both TTF and usage intentions. Notably, performance and effort expectancies positively drive usage intentions; conversely, facilitating conditions exert a negative impact, and social influence demonstrates no significant effect in this context. This study offers robust theoretical contributions by synthesizing the UTAUT and TTF frameworks and provides actionable insights for practitioners and policymakers to promote smart home adoption and support sustainability objectives.

## Introduction

 Smart homes, with their built-in smart home appliances, have thus become an indispensable part of a smart city^1–3^. With the burgeoning development of IoT services and technological solutions for the home, smart home appliances have gained widespread popularity on the market and are widely accepted by users^1^. Usage behaviors of smart home appliances refer to how residents choose to use smart home appliances and their automatic control functions to obtain an environment for smart energy use and fulfill the potential for energy savings and carbon reduction potential^4^. Since the development of smart home appliances is not completely new, their current pace of development falls short of expectations^5^. This may be the result of influencing factors, including lifestyle habits, reliability, fear of technical complexity, privacy and security risks, and maintenance costs^1,5,6^. As the development of the smart home appliance market needs to be sustained by consumers’ purchasing behaviors^7^, understanding consumers’ usage intentions can help with better predicting the future penetration rates of such appliances.

Research exploring factors influencing users’ adoption of smart home appliances is a relatively new area of study^2^, with most studies examining usage intentions based on technology acceptance theories, such as the technology acceptance model (TAM), value-based adoption model (VAM), theory of planned behavior (TPB), and value-belief-norm (VBN) theory^2,4,8^. TAM, which merely deals with users’ short-term attitudes and beliefs before or after acceptance^9^, is insufficient to fully address characteristics in complex environments, as it lacks the constructs crucial for understanding users’ usage intention of advanced technology^10^. The unified theory of acceptance and use of technology (UTAUT) combines various theoretical models and constructs to overcome the abovementioned limitation and enhance its explanatory power^11^. Therefore, using UTAUT as the primary framework for predicting users’ behavioral intentions can significantly improve the previously known limitations of TAM. UTAUT is also considered one of the most influential and comprehensive frameworks for predicting the acceptance and adoption of technology^12,13^, and it has been proven to have good predictability^11–13^. That being said, few studies have applied the UTAUT model to explore the usage intention of smart home appliances.

Task-technology fit (TTF) is a theoretical framework that has been widely studied and applied to various information systems studies^8^ and is generally used to evaluate how information technology is linked to performance. This theory postulates that the match between task characteristics and technical characteristics contributes to better technology acceptance by helping individuals accomplish tasks more effectively^14,15^. The TTF construct measures the extent to which a certain technology helps an individual perform tasks. In the context of smart home appliances, users who believe that technical characteristics (e.g., the functionality of a smart home appliance) are unable to match the task needs are more likely to abandon the technology. Whether or not such appliances can satisfy consumers’ actual needs is also a key determinant of public acceptance. Based on these arguments, this study infers that users’ adoption of smart home appliances may be determined not only by how they view the technologies involved, but also by whether the task-technology fit is good. However, as far as we are aware, few studies have expounded on consumers’ usage intention of smart home appliances from the perspective of user perceptions and TTF. Therefore, the current study recommends expanding the research framework to use TTF as a reinforcing factor for explaining and predicting consumer behaviors.

Although the UTAUT theoretical framework has its merits, it still requires further theoretical testing using other factors to validate its applicability to user behaviors under different circumstances^11^. According to the innovation diffusion theory, users’ innovativeness is a good explanatory factor for innovation adoption^16^. Hence, personal innovativeness (PI) was added to test the further applicability and explanatory power of the model^9,17^. Source credibility is a significant heuristic cue closely related to the persuasiveness of information^18,19^. Perceived source credibility is regarded as the extent to which information users perceive an information source as reliable, competent, and trustworthy^20,21^. If a source appears expert and trustworthy, it exerts a stronger influence and may further impact users’ behaviors^18,20,21^. Trust is a relationship of reliance with applications in a wide range of areas. In the field of marketing, it is believed to be an indispensable component of all business transactions, and it often becomes a key focus of studies on the adoption of new technology^22,23^. Users’ trust in smart home appliances plays a fundamental role in reducing uncertainty regarding smart home adoption. To better explain factors related to users’ choice of technology, the TTF model was extended using attitude/behavior models such as TAM and UTAUT^9,10,24^. The results showed that the TTF model produced a more comprehensive explanation of usage intentions and the rate of technology utilization when used in conjunction with other theories rather than alone^9,10^.

Regarding the above, the current study aims to fill the gap in existing research. The purpose is to analyze the key factors influencing consumers’ usage intention of smart home appliances by integrating the UTAUT and TTF models and extending their scope to include trust, perceived source credibility, and personal innovativeness as the other influencing factors. We chose to include UTAUT because it is considered to be one of the most comprehensive models for explaining technology acceptance, and it reflects users’ perceptions of technology, such as performance expectancy and effort expectancy. Meanwhile, when evaluating the fit between tasks and technology, we believed that combining TTF with UTAUT would likely explain a higher level of variance in consumer acceptance than using TTF alone.

Specifically, the following research questions were formulated: (1) What are the outcomes of the effects of perceived source credibility and trust? (2) How does TTF affect consumers’ usage intentions in the context of smart home appliances? (3) What are the outcomes of the effects of personal innovativeness on TTF and usage intentions? (4) How do the four variables in the UTAUT model affect consumers’ usage intention of smart home appliances? This study had four main purposes: (1) To understand the effects of perceived source credibility and trust on performance expectancy; (2) To examine the effect of TTF on the UTAUT model; (3) To investigate the effects of personal innovativeness on TTF and usage intentions; and (4) To explore the effects of the four variables in the UTAUT model on usage intentions. Lastly, the contributions of this study can be summarized as follows: (1) It focuses specifically on perceived source credibility and trust, which play a fundamental role in the area of smart home consumption; (2) It advances theoretical understanding by repositioning TTF as a cognitive appraisal mechanism that activates all four UTAUT constructs, forming a TTF-to-UTAUT mediation pathway; and (3) It reveals that personal innovativeness plays a significant role in the area of smart home appliances.

This study advances the theoretical understanding of technology adoption by repositioning TTF as a cognitive appraisal mechanism that activates users’ evaluative judgments across all four UTAUT constructs, rather than serving as a parallel predictor. The resulting TTF-to-UTAUT mediation pathway offers a mechanistic explanation for how task-technology alignment shapes performance expectancy, effort expectancy, social influence, and facilitating conditions, which in turn drive usage intentions. Personal innovativeness further extends this framework by operating at both the appraisal stage (TTF) and the behavioral stage (usage intention), revealing the multi-level influence of individual difference variables in technology adoption.

## Literature review and research hypotheses

### Smart home appliances

As the integration of IoT technology (e.g., Wi-Fi), AI, and mobile Internet technology continues to deepen, smart home appliances will be making inroads into family life. The widespread adoption of these appliances will offer people more convenient personalized home services and help save energy and reduce carbon emissions^1,2^. A smart home is a cyber-physical system equipped with interconnected sensors that uses the Internet to control, collect, process, and store information, interact with its environment, and communicate with other devices. Meanwhile, appliances with an embedded IT system that controls a variety of functions and can make decisions are referred to as smart home appliances^1,4,8^. Examples include smart TVs, smart refrigerators, smart washing machines, smart lighting, and smart speakers. These appliances are an integral part of the complete network of smart home services and devices^1,2,4,8^. They introduce sensor and network communications technologies into the conventional home, enabling it to sense and respond to the living environment automatically. For example, smart lighting systems can automatically turn on or off and adjust their brightness upon sensing the presence of individuals in the room and the outdoor lighting environment to avoid wastage of electricity due to forgetting to turn the lights off. Smart refrigerators, on the other hand, can regulate the temperature of the freezer compartment automatically based on the quantity of items inside and lower it to a suitable level if the contents require a lower temperature in order to keep food fresh and reduce energy consumption. Therefore, previous scholars^25,26^ argued that the deployment of smart homes is an important element in the transition toward a low-carbon economy, thereby achieving environmental sustainability goals.

### Task-technology fit (TTF)

The theory of TTF is a concept that evaluates the extent to which technology can fulfill certain task requirements^14^. It postulates that the match between task requirements and technological functionality affects utilization rate and performance and posits that task characteristics and technology characteristics are two key antecedents of TTF^9,15^. Task characteristics refer to the actions performed by individuals to convert inputs into outputs, whereas technology characteristics are defined as the tools they use when performing tasks. Both types of characteristics exert an influence on TTF, which consequently affects the performance and utilization rate of technology users^9,10^. As previous studies on information technology have substantiated the positive correlation between task characteristics, technology characteristics, and TTF^27–29^, the current study hypothesizes the following:

H1: Task characteristics have a significant influence on TTF.

H2: Technology characteristics have a significant influence on TTF.

### Unified theory of acceptance and use of technology (UTAUT)

Before UTAUT was proposed, several theories attempted to explain the underlying reason for individuals’ use of information systems^27,30,31^. Nevertheless, these theories had limitations in fully explaining the use of technology, as they only covered certain aspects and often overlapped in their explanations^11,12,27,32^. Many researchers recognized the need for a comprehensive framework that could explain the significance of users’ technology acceptance. To address this need, Venkatesh et al.^11^ developed UTAUT by combining eight existing theories and models, resulting in a theoretical model that outperformed any of the eight existing ones in terms of predictive power^11–13^. The eight models are as follows: (1) Theory of Reasoned Action (TRA)^33^, (2) Theory of Planned Behavior (TPB)^34^, (3) Technology Acceptance Model (TAM)^35^, (4) Motivational Model (MM)^36^, (5)Social cognitive theory (SCT)^37^, (6) Model of Personal-Computer Utilization (MPCU)^38^, (7) a combination of the TAM and TPB (C-TAM-TPB)^39^, and (8) Innovation diffusion theory (IDT)^40^.

UTAUT identifies four determinants that influence users’ usage intention of information systems, namely, performance expectancy, effort expectancy, social influence, and facilitating conditions^11^. Performance expectancy (PE) is described as the extent to which users believe that using new technology will help improve their performance in everyday activities^11,13,32^ proved that PE is an important predictor of consumers’ behavioral intentions. Effort expectancy (EE) represents users’ perception of ease associated with the learning and use of new technology^11^; users are generally willing to use new technology if it is easy to use. Social influence (SI) expresses individuals’ perception of the extent to which others important to them (e.g., family members and friends) agree with their specific behaviors^11^. Facilitating conditions (FC) refer to individuals’ perception of the extent to which an organizational and technical infrastructure supports the use of the system^11,30^. Previous research demonstrated that increased FC would augment users’ behavioral intentions toward technology acceptance. Apart from the above, UTAUT also consists of moderating (or indirect) effects (i.e., age, gender, experience, and voluntariness of use)^11^.

### Combination of UTAUT and TTF

Combining the TTF model, which helps explain user acceptance from the perspective of task-technology fit, with the UTAUT model, which emphasizes users’ perception of technology, may generate a powerful theoretical framework that can increase the variance in the behavioral intention to adopt new technology and help explore and understand users’ adoption of new technology^27,32,41^. According to previous studies, by integrating these two models to predict the use of technology valuable attributes can be obtained for analysis^27^. The models were integrated for several reasons: (1) Combining UTAUT with TTF can enhance the explanatory power for usage intention; (2) Previous research has showed that UTAUT and TTF are highly correlated and enhance the use of new technology; and (3) In addition to the determinants included in the UTAUT model, many other factors can be used to explain consumers’ use of smart home appliances^27,32^.

Smart home appliances represent a unique adoption context where TTF plays a particularly critical role. Unlike enterprise information systems where organizational mandates often drive adoption, smart home appliances require voluntary adoption by individual consumers who must evaluate whether the technology aligns with household-specific tasks (e.g., energy management, convenience, safety). This context-specific characteristic makes the TTF-to-UTAUT pathway especially salient: consumers first assess whether the appliance fits their domestic task requirements, and this assessment subsequently shapes their perceptions of performance, ease of use, social acceptability, and available support.

Most of the studies on UTAUT and TTF^9^ shed light on the impact of the UTAUT model (performance expectancy, effort expectancy, social influence, facilitating conditions) on TTF or that of TTF on performance expectancy^27,28^. However, Park et al.^24^ regarded TTF as a crucial external variable for UTAUT, influencing behavioral intentions. The results indicated that TTF had a significantly positive impact on performance expectancy, effort expectancy, social influence, facilitating conditions, and usage intention. Based on previous research, this study hypothesizes that consumers’ perceived TTF positively influences each of the four constructs of UTAUT. Based on the above, we proposed the following hypotheses:

H3: TTF has a positive influence on performance expectancy.

H4: TTF has a positive influence on effort expectancy.

H5: TTF has a positive influence on social influence.

H6: TTF has a positive influence on facilitating conditions.

Previous research has suggested that comprehensively exploring the impact of TTF on usage intention of technology is crucial across a variety of fields and that TTF is directly associated with usage intention^9,15,27,41^. Hence, this study further delved into the effects of TTF on consumers’ usage intention of smart home appliances. Based on previous studies, it hypothesized that TTF directly affects the usage intention of smart home appliances.

H7: TTF has a positive influence on usage intention.

UTAUT is a theory that has been widely adopted and applied to a variety of settings, such as the metaverse, revolutionary technology-driven products (RTP), and mobile health (mhealth) services. Sediyaningsih et al.^27^ conducted a study to explore the factors influencing usage intentions toward metaverse technology in digital library services based on the context of higher education and found that usage intentions were significantly affected by performance expectancy and social influence. Moreover, Park et al.^24^ examined factors affecting consumers’ usage intention of a revolutionary technology-driven product (RTP) and found that while performance expectancy and social influence had a significantly negative impact on usage intentions, effort expectancy and facilitating conditions had no influence on the same. In a study exploring factors that affected patients’ use of mHealth services in a developing country (Bangladesh), Alam et al.^12^ expanded the UTAUT model by incorporating perceived reliability and price value. The results demonstrated that the behavioral intention to use mHealth services was positively affected by performance expectancy, social influence, facilitating conditions, and perceived reliability, whereas price value and effort expectancy had no significant influence on behavioral intentions. As UTAUT identifies performance expectancy, effort expectancy, social influence, and facilitating conditions as key antecedents that affect technology acceptance^11,12,27,30,32^, this study argues that consumers’ performance and effort expectancy regarding smart home appliances as well as social influence and facilitating conditions are crucial antecedents that influence usage intentions. Therefore, we hypothesized the following:

H8: Performance expectancy has a significant influence on the usage intention of smart home appliances.

H9: Effort expectancy has a significant influence on the usage intention of smart home appliances.

H10: Social influence has a significant influence on the usage intention of smart home appliances.

H11: Facilitating conditions have a significant influence on the usage intention of smart home appliances.

### Trust

Trust has repeatedly been recognized as one of the major drivers of successful technology adoption and a key factor for addressing uncertain behavioral characteristics^2,22,23,42^. In the context of smart home appliances, the trust users place in appliances can help overcome concerns over the associated risks and reduce the uncertain factors relating to the use of such appliances^2,23^. Given the widespread application of trust across various fields, its definitions vary extensively. Liu et al.^23^ defined trust as users’ confidence in the reliability of smart home appliances to meet their needs and expectations, while Shuhaiber and Mashal^2^ defined it as users’ confidence in the reliability of smart homes to meet their expectations for the system. In this study, we define trust as users’ confidence in the reliability of smart home appliances to meet their needs and expectations.

Several studies have investigated the relationship between trust and TAM. For instance, Zhang^22^ has pointed out that trust exerts an influence on perceived usefulness, perceived ease of use, and behavioral intention, whereas Tang and Jiang^42^ have established it as a predictor of perceived usefulness. However, most previous studies explored the impact of UTAUT (i.e., performance expectancy, effort expectancy, social influence, and facilitating conditions) on trust^41,43^, and the direct effect of trust on performance expectancy has not been fully explored. Trust, defined as users’ confidence in the reliability and functional capability of smart home appliances, conceptually aligns most closely with performance expectancy, which captures expected functional benefits. This conceptual mapping is more direct than the relationships between trust and effort expectancy (ease of use), social influence (others’ opinions), or facilitating conditions (resource availability). Therefore, this study proposes that trust is a predictor of performance expectancy. Specifically, we hypothesized that:

H12: Trust has a significant influence on performance expectancy.

### Perceived source credibility

The source credibility theory relates to the reputation of information sources and postulates that individuals tend to agree with respectable sources^44^. The theory also maintains that individuals or information recipients may quickly switch to accepting communications from a source when it presents itself as credible. Perceived source credibility refers to the extent to which information users perceive an information source to be reliable, competent, and trustworthy^20,21^. Liu et al.^20^ defined perceived source credibility as the credibility of an mHealth service as perceived by recipients. Similarly, the current study defines it as the extent to which users perceive the energy-saving performance claims associated with smart home appliances to be credible and trustworthy.

According to the source credibility theory, more credible sources tend to be more persuasive and are likely to engender more positive attitudes and behaviors than less credible ones^18,19,44^. Bhattacherjee and Sanford^21^ pointed out that source credibility is an important factor influencing perceived usefulness and attitude. Liu et al.^20^ also identified perceived source credibility as a predictor of perceived usefulness. Previous studies largely focused on exploring the impact of perceived source credibility on perceived usefulness. However, perceived usefulness is a measurement variable of the TAM model that bear similarities with performance expectancy in the UTAUT model. Source credibility theory posits that credible sources primarily influence recipients’ beliefs about the efficacy and utility of a recommended behavior^21^. This aligns with performance expectancy rather than with effort expectancy, social influence, or facilitating conditions. As the impact of perceived source credibility on performance expectancy warrants further investigation, we propose that perceived source credibility is a predictor of performance expectancy and, specifically, hypothesize the following:

H13: Perceived source credibility has a significant influence on performance expectancy.

### Personal innovativeness (PI)

Innovativeness refers to an individual’s inclination or intention toward being the first to use new technology^17,32^, and it can become a source of motivation for individuals to use technology^45^. According to the innovation diffusion theory, users’ innovativeness is an attribute that explains innovation adoption well^16^. Innovativeness concerns the degree to which an individual adopts an innovation more quickly than others^46^, and it amounts to individuals’ usage intention of new information technology^47^. Kim et al.^9^ also defined personal innovativeness as individuals’ usage intention of new technology. In the current study, personal innovativeness is defined as an individual’s willingness to experiment with and adopt new technologies ahead of others.

Previous research has indicated that innovation characteristics are key factors that affect the adoption and use of new technology^17,32^. This study attempts to expand this model by adding personal innovativeness (PI). Among previous studies that explored the relationship between PI and technology adoption behaviors, various papers on new technology adoption highlighted a significant viewpoint that consumers’ innovativeness affected usage intentions in different contexts^17,32^. Kim et al.^9^ stated that, while personal innovativeness was the strongest predictor of consumers’ intention to use BOPS (Buy Online & Pick up in Store), it did not affect TTF. With reference to previous research^9,16^, the current study predicts that higher levels of personal innovativeness are associated with higher perceived TTF and usage stronger intentions of smart home appliances. Based on the discussion above, we hypothesize that:

H14: Personal innovativeness has a significant influence on TTF.

H15: Personal innovativeness has a significant influence on the usage intentions of smart home appliances.

**Fig. 1:**
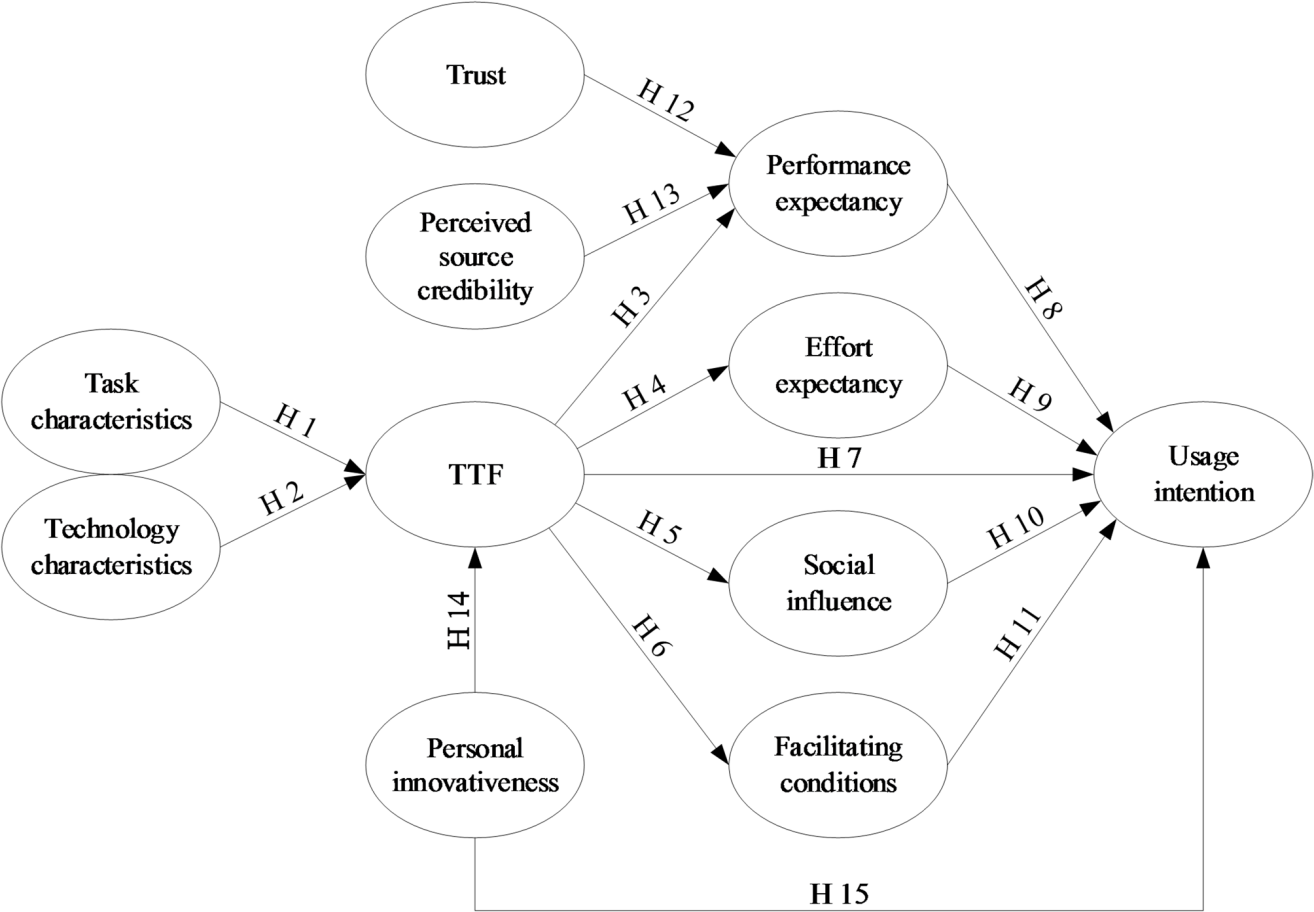
The proposed research model.

To understand the determinants influencing consumers’ usage intention of smart home appliances, this study integrated the UTAUT and TTF models and included trust, perceived source credibility, and personal innovativeness. The research framework thus proposed in this study is illustrated in Fig. 1, with arrows denoting the hypothesized relationships between variables.

## Methodology

### Measurement items and questionnaire design

The questionnaire survey comprised three sections. The first section was an introduction to the research background, including the concept of smart home appliances and relevant information, to ensure that the respondents shared a level of understanding about smart home appliances. This section also included the study purposes and an obligatory guarantee of confidentiality for questionnaire responses, which stated, while encouraging respondents to answer honestly, that their personal information would be fully protected and not be used for any purpose other than that required for the specified study. The second section gathered information about demographic statistics, such as the respondent’s gender, age, monthly income, and formal education. In the third section, all the measurement items were scored on a five-point Likert-type scale ranging from 1 = “strongly disagree” to 5 = “strongly agree.” To ensure that each respondent was paying close attention to the questions when participating in the survey, this section also included attention check questions; respondents who failed to answer these questions correctly would be disqualified from the survey.

In terms of the design of the measurement items for each research variable, first, the items measuring the principal constructs of the UTAUT model, namely, performance expectancy (5 items), effort expectancy (5 items), social influence (5 items), facilitating conditions (5 items), and usage intention (3 items), were mainly adapted from questionnaires developed by previous scholars^11–13,24,27,32^. Second, the items measuring the constructs of the TTF model, namely, task characteristics (5 items), technology characteristics (4 items), and TTF (3 items), were also largely adapted from questionnaires designed by previous scholars^9,15,24,28^. The questionnaire design for trust (4 items) was modified from measurement items developed by Zhang^22^, Liu et al.^23^, and Tang and Jiang^42^, and that for perceived source credibility (3 items) was revised and adopted from previous scholarly work by previous scholars^18,19,44^. Lastly, personal innovativeness (5 items) was measured using items adapted from questionnaires developed in previous studies^9,17,32^. To sum up, the research framework constructed in this study comprised nine research variables and 47 measurement items. There were also four items pertaining to the respondents’ demographic information, namely, their gender, age, monthly income, and formal education. All measurement items in this study are shown in Table 5 (Appendix).

The questionnaire survey was prepared and completed after extensively consulting the literature. The first draft was developed in English, followed by translation into Chinese by five professionals who were well-versed in both English and Chinese. The researchers compared and revised the translations of various questionnaires to ensure that the items in the final version were articulated clearly in Chinese. The questionnaire was then back-translated into English to ensure accuracy and consistency between the translation and the original. Before collecting questionnaire data on a large scale, the survey was pre-tested by 10 respondents, who were asked to complete the questionnaire and comment on its clarity and validity. The questionnaire was then refined based on their feedback.

Following the above steps, 78 respondents were selected across the Taichung region of Taiwan for a pilot test in January 2024 to evaluate the reliability of the measurement outcomes. A Cronbach’s alpha analysis showed that, in general, each construct scored above the recommended minimum threshold of 0.7 (with the coefficients ranging between 0.783 and 0.937)^48^. Therefore, we concluded that all the variables in this study had good reliability and were acceptable. Eventually, the actual data collection process was performed using the modified version of the questionnaire.

### Survey design

The main empirical study was carried out from March to April 2024 by means of an online survey, social media tools, and a face-to-face questionnaire survey. Prior to each questionnaire survey, respondents were informed of the research purposes either in writing or verbally, that the questionnaire would be conducted anonymously, and that their responses would be kept in absolute confidentiality and used for the sole purpose of academic research. They were also told that they were free to stop answering and withdraw from the survey immediately should they experience any discomfort or concern during the survey. Based on the selection criteria, 186 respondents were removed from the initial pool of 2,105, resulting in a final sample of 1,919 respondents, excluding the participants of the pilot test. Table 1 presents a summary of the demographic statistics of the respondents. The 1,919 respondents comprised 51.5% males and 48.5% females, respectively. In addition, respondents aged between 40 and 49 years accounted for 46.0% of the overall sample; while those aged 39 or below comprised 22.6%; between 50 and 59, roughly 22.1%; and 60 or above, 9.3%. Lastly, approximately 40.7% of the respondents were college or university graduates.

**Table 1:** Demographic details of respondents. (*N* = 1,919).

Factor/Level	*N*	%	Factor/Level	*N*	%
*Gender*			*Age*		
Male	989	51.5	≦ 39	434	22.6
Female	930	48.5	40–49	883	46.0
*Monthly Income (TWD)*			50–59	424	22.1
< 20,000	369	19.2	≧ 60	178	9.3
20,001–40,000	1039	54.1	*Formal Education*		
40,001–60,000	362	18.9	Elementary school and below	202	10.5
60,001–80,000	101	5.3	Junior high school	201	10.5
> 80,001	48	2.5	Senior/vocational high school	571	29.8
			College and university	781	40.7
			Graduate institute and above	164	8.5

### Non-response bias (NRB) and common method bias (CMB)

Non-response bias (NRB) occurs when there are systematic differences between individuals who respond to a survey and those who are invited to participate in it but do not respond^49^, which may hinder the generalizability of research results. Armstrong and Overton^50^ recommended dividing the data into two subsets (early and late respondents) to ensure that NRB would not be a concern for the study. The results of analysis revealed no significant difference between the early and late respondents (*p* > 0.05), indicating that the present study was not impacted by NRB. Meanwhile, Podsakoff, MacKenzie, Lee, and Podsakoff^51^ stated that common method bias (CMB) is a major issue faced by researchers when using survey methods. Podsakoff et al.^51^ recommended employing Harmon’s single-factor test to address the problem of CMB^51,52^. The analytical results indicated that the first factor accounted for 42.75% (compared to a recommended value of < 50%) of the total explained variance. Hence, CMB was not an issue of concern in this study.

## Analytical results

Structural equation modeling (SEM) was applied for data analysis for the following reasons: (1) SEM is an effective method used in exploratory research or theory development; (2) It is suitable for analyzing complex models involving multiple research variables and indicators; (3) It is a statistical procedure that includes testing the measurements and predictive and causal hypotheses; and (4) The modelling process accounts for errors of measurement, thereby improving the accuracy of model estimates^53,54^. Considering the presence of formative constructs in SEM as well as factors related to the nature of the collected data, the predictive aspect of the study, the complexity of the hypothesized model, and the like, partial least squares structural equation modeling (PLS-SEM) was employed to test the hypothesized model^55^.

According to the recommendation of Hair et al.^48^, this study adopted a two-stage data analysis method to test the research model during the SEM analysis. To evaluate the validity and reliability of the variables constructed in this study, confirmatory factor analysis (CFA) was first conducted to assess the measurement model. The structural model was then analyzed using the bootstrapping procedure with 5,000 iterations to verify each of the hypotheses proposed in this study.

### Measurement model analysis

The reliability and validity of the research model were first evaluated to ensure the consistency of data. Reliability analysis usually relies on the Cronbach’s alpha coefficient, whereas the validity and internal consistency of the model are typically tested using CFA. In addition, this study demonstrates skewness and kurtosis^56^. The test results of the model in this study are presented in Table 2.

The Cronbach’s alpha values ranged between 0.603 and 0.962, largely exceeding the 0.7 threshold^48,57^ and indicating relatively high reliability. Furthermore, previous researchers^48,57^ have suggested that, in order for convergent validity to be established, the factor loadings must exceed 0.7, whereas the composite reliability (CR) and average variance explained (AVE) must be greater than 0.7 and 0.5, respectively. The factor loadings, CR values, and AVE values of all the measurement items exceeded the recommended thresholds, indicating that all metrics met the criteria. Thus, this study satisfied all the conditions for convergent validity, lending support for its convergent validity.

**Table 2:** Construct reliability results.

Construct	Items	Item loading	Mean	S.D.	Skewness	Kurtosis	Cronbach’s α	rho_A	CR	AVE
TASC	TASC1	0.853	3.87	0.63	−0.54	0.97	0.915	0.918	0.936	0.746
	TASC2	0.886	3.88	0.63	−0.08	−0.09				
	TASC3	0.874	3.73	0.65	−0.20	0.02				
	TASC4	0.870	3.75	0.69	−0.11	−0.19				
	TASC5	0.833	3.83	0.83	−1.41	3.11				
TECC	TECC1	0.856	3.71	0.61	0.26	−0.63	0.882	0.938	0.916	0.732
	TECC2	0.867	3.60	0.73	−0.28	−0.16				
	TECC3	0.875	3.68	0.67	−0.44	0.23				
	TECC4	0.823	3.65	0.67	−0.17	−0.09				
TTF	TTF1	0.908	3.95	0.58	−0.10	0.16	0.839	0.863	0.902	0.755
	TTF2	0.836	3.93	0.57	−0.17	0.45				
	TTF3	0.861	3.69	0.69	−0.25	0.75				
TRU	TRU 1	0.789	3.69	0.70	−0.35	0.08	0.874	0.953	0.910	0.718
	TRU 2	0.810	3.68	0.63	0.22	−0.56				
	TRU 3	0.911	3.73	0.63	−0.21	−0.01				
	TRU 4	0.874	3.82	0.60	−0.45	0.41				
PSC	PSC1	0.962	3.90	0.64	−0.24	0.29	0.925	0.957	0.951	0.867
	PSC2	0.898	3.84	0.58	−0.35	0.49				
	PSC3	0.933	3.48	0.77	−0.34	0.64				
PI	PI1	0.881	3.25	0.86	−0.31	−0.43	0.936	0.944	0.951	0.795
	PI2	0.867	3.35	0.81	−0.06	−0.45				
	PI3	0.915	3.53	0.74	−0.11	0.03				
	PI4	0.913	3.57	0.76	−0.02	−0.30				
	PI5	0.881	3.91	0.66	−0.63	0.79				
PE	PE1	0.904	3.84	0.64	−0.52	0.83	0.929	0.931	0.946	0.779
	PE2	0.896	3.97	0.58	−0.51	0.77				
	PE3	0.894	3.97	0.57	−0.50	1.53				
	PE4	0.853	4.00	0.58	−0.19	0.68				
	PE5	0.864	3.79	0.70	−0.39	1.31				
EE	EE1	0.849	3.78	0.69	−0.22	−0.05	0.930	0.938	0.947	0.780
	EE2	0.902	3.83	0.69	−0.42	1.11				
	EE3	0.866	3.67	0.72	−0.41	1.12				
	EE4	0.896	3.69	0.73	−0.16	−0.20				
	EE5	0.902	3.59	0.64	−0.25	−0.11				
SI	SI1	0.603	3.66	0.79	−0.66	0.15	0.805	0.825	0.863	0.560
	SI2	0.772	3.65	0.75	−0.95	1.95				
	SI3	0.784	4.02	0.53	−0.97	2.47				
	SI4	0.783	3.81	0.60	0.02	0.54				
	SI5	0.785	3.58	0.67	−0.06	−0.08				
FC	FC1	0.800	3.76	0.66	−0.47	−0.01	0.905	0.916	0.929	0.724
	FC2	0.828	3.67	0.71	−0.46	0.43				
	FC3	0.860	3.64	0.72	−1.44	3.31				
	FC4	0.918	3.73	0.69	−0.61	1.06				
	FC5	0.844	3.64	0.68	−0.32	0.10				
UI	UI1	0.951	3.64	0.64	−0.38	0.06	0.889	0.909	0.931	0.820
	UI2	0.934	3.65	0.69	−0.33	0.04				
	UI3	0.826	3.87	0.63	−0.11	−0.17				

The Fornell-Larcker criterion and the value of heterotrait-monotrait ratios (HTMT) were also used to test the discriminant validity. Table 3 outlines the resulting coefficients of HTMT and related analyses. The analytical results showed that all the constructs had an HTMT coefficient smaller than 0.85^58^ and the square root of the AVE for each construct was higher than its correlation coefficient with all other constructs. In other words, the model demonstrated strong discriminant validity.

**Table 3:** Correlations among major constructs.

1. TASC	Mean	S.D.	1.	2.	3.	4.	5.	6.	7.	8.	9.	10.	11.
	3.81	0.59	0.864	0.440	0.711	0.576	0.495	0.411	0.548	0.678	0.644	0.609	0.489
2. TECC	3.66	0.58	0.496	**0.856**	0.427	0.701	0.581	0.561	0.382	0.518	0.507	0.591	0.488
3. TTF	3.91	0.49	0.811	0.504	**0.869**	0.624	0.657	0.408	0.474	0.478	0.393	0.506	0.564
4. TRU	3.70	0.57	0.652	0.803	0.730	**0.848**	0.641	0.538	0.458	0.591	0.338	0.573	0.624
5. PSC	3.85	0.57	0.542	0.649	0.742	0.714	**0.931**	0.436	0.530	0.417	0.302	0.482	0.552
6. PI	3.44	0.70	0.453	0.621	0.465	0.598	0.472	**0.892**	0.628	0.449	0.358	0.476	0.661
7. PE	3.94	0.54	0.598	0.427	0.538	0.512	0.575	0.352	**0.882**	0.602	0.460	0.502	0.540
8. EE	3.75	0.62	0.736	0.575	0.544	0.657	0.449	0.483	0.647	**0.883**	0.534	0.729	0.488
9. SI	3.75	0.50	0.752	0.594	0.499	0.416	0.351	0.414	0.549	0.630	**0.749**	0.612	0.347
10. FC	3.68	0.59	0.672	0.660	0.584	0.643	0.528	0.518	0.546	0.795	0.728	**0.851**	0.399
11. UI	3.65	0.58	0.554	0.564	0.652	0.711	0.610	0.729	0.595	0.536	0.427	0.449	**0.905**

Note: The diagonal elements in bold represent the square roots of the AVE for each construct, the values below are the HTMT values, and those above are the correlation coefficients between variables.

### Structural model analysis

The hypothesized relationships were evaluated using SEM. To gauge the significance of the path coefficients, 5,000 subsamples were used for a bootstrap test. The R^2^ values of the endogenous variables exceeded 0.1^59^, indicating that the conceptual model could explain a substantial portion of the variance in purchase intentions toward smart home appliances (R^2^ = 0.619). Additionally, the model-explained variance for TTF was 55.6%; PE, 34.6%; EE, 25.1%; SI, 20.0%; and FC, 27.5%.

**Fig. 2:**
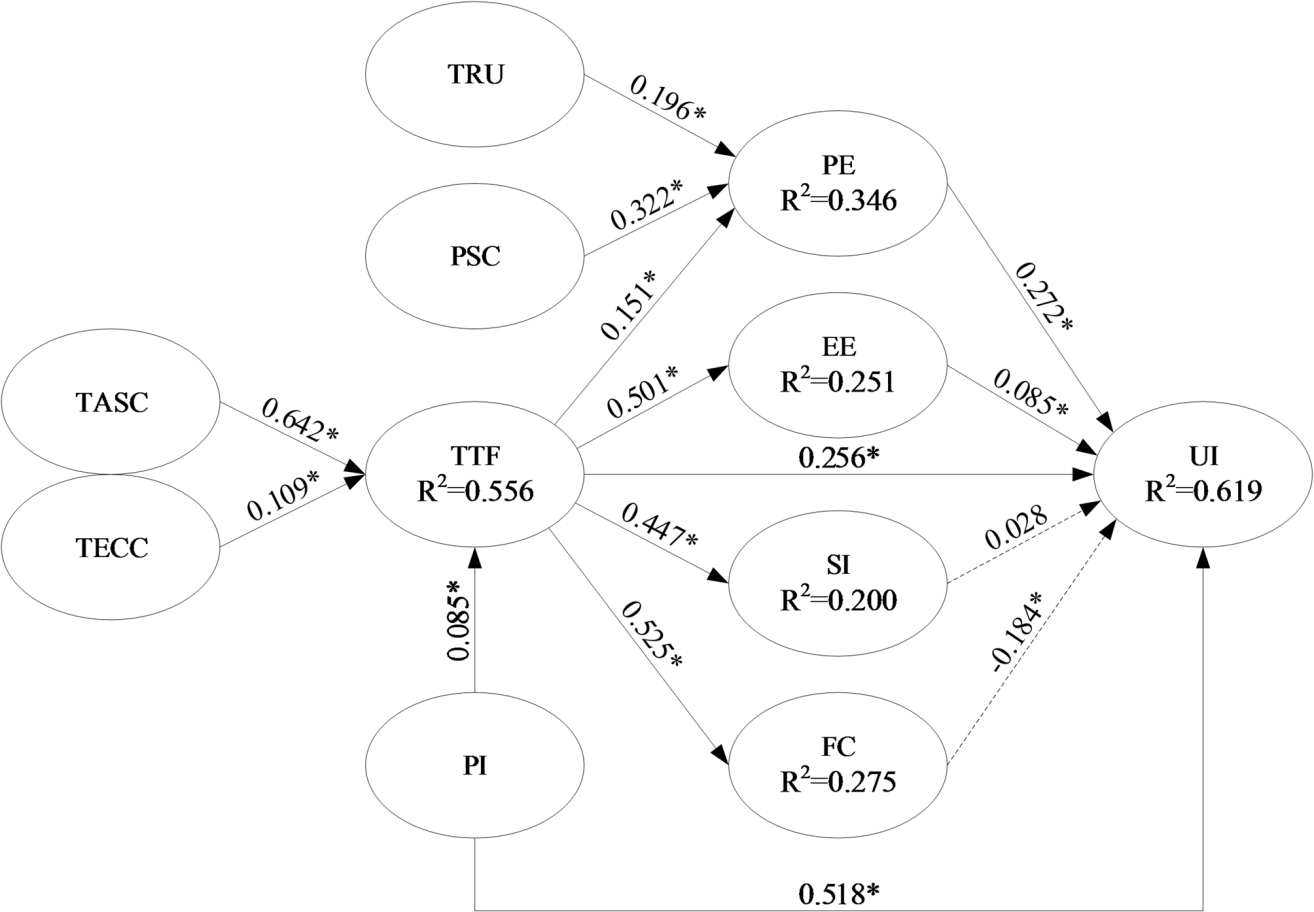
Empirical results of the structural path model. Value on path: standardized coefficients (β), R^2^: Coefficient of determination, and *p < 0.05.

As illustrated in Table 4, TASC (H1, β = 0.642, t = 41.685) and TECC (H2, β = 0.109, t = 5.409) had a significantly positive influence on TTF; hence, H1 and H2 were substantiated. Since TTF was also shown to exert a significantly positive influence on PE (H3, β = 0.151, t = 5.677), EE (H4, β = 0.501, t = 27.872), SI (H5, β = 0.447, t = 26.466), FC (H6, β = 0.525, t = 30.164), and UI (H7, β = 0.256, t = 11.193), H3–H7 were validated. In the UTAUT model, PE (H8, β = 0.272, t = 12.279) and EE (H9, β = 0.085, t = 3.092) had a significantly positive influence on UI; hence, H8 and H9 were supported. However, SI (H10, β = 0.028, t = 1.247) had no significant influence on UI, while FC (H11, β= −0.184, t = 8.388) had a significantly negative influence on UI; therefore, H10 and H11 were rejected. Lastly, in terms of the extended variables, TRU (H12, β = 0.196, t = 7.295) and PSA (H13, β = 0.322, t = 10.374) had a significantly positive influence on PE, which supported H12 and H13. PI was also found to have a significantly positive effect on TTF (H14, β = 0.085, t = 3.703) and UI (H15, β = 0.518, t = 21.987); thus, H14 and H15 were supported. The results of hypothesis testing for the research model are presented in Table 4; Fig. 2.

**Table 4:** Hypothesis testing outcomes of the structural path model.

Hypothesis	Relationships between variables	Standardized coefficient	t-statistic	Test results
H1	TASC → TTF	0.642*	41.685	Supported
H2	TECC → TTF	0.109*	5.409	Supported
H3	TTF → PE	0.151*	5.677	Supported
H4	TTF → EE	0.501*	27.872	Supported
H5	TTF → SI	0.447*	26.466	Supported
H6	TTF → FC	0.525*	30.164	Supported
H7	TTF → UI	0.256*	11.193	Supported
H8	PE → UI	0.272*	12.279	Supported
H9	EE → UI	0.085*	3.092	Supported
H10	SI → UI	0.028	1.247	Not supported
H11	FC → UI	−0.184*	8.388	Not supported
H12	TRU → PE	0.196*	7.295	Supported
H13	PSC → PE	0.322*	10.374	Supported
H14	PI → TTF	0.085*	3.703	Supported
H15	PI → UI	0.518*	21.987	Supported

**Table 5:** Measurement Items.

Construct		Items
TASC	TASC1	I need smart appliances to save energy.
	TASC2	I believe that smart appliances can meet my energy-saving needs.
	TASC3	I think smart appliances are easy to use.
	TASC4	I believe that smart appliances can save energy at any time.
	TASC5	Smart appliances are important to my daily life.
TECC	TECC1	Smart appliances can save energy everywhere.
	TECC2	Smart appliances offer instant services.
	TECC3	Smart appliances keep me safe.
	TECC4	Smart appliances can improve energy-saving efficiency.
TTF	TTF1	I think smart appliances can help me save energy.
	TTF2	I believe that smart appliances are safe for energy-saving activities.
	TTF3	Overall, I think smart appliances fully meet my needs for energy-saving activities.
TRU	TRU1	I believe that smart appliances can protect my privacy.
	TRU2	I believe that smart appliances are reliable.
	TRU3	I believe that smart appliances are trustworthy.
	TRU4	In general, I can trust smart appliances.
PSC	PSC1	I believe that I can save energy by using smart appliances.
	PSC2	I believe that the energy-saving efficiency provided by smart appliances is very important and helpful.
	PSC3	I believe that the energy-saving efficiency provided by smart appliances is trustworthy.
PI	PI1	When I hear about new technology, I try to work out how to use it.
	PI2	Among my peers, I am usually the first to try new information technology.
	PI3	I like to try new information technology.
	PI4	I am more open to innovative ideas than my friends are.
	PI5	When using technology, I encounter fewer problems than others do.
PE	PE1	I think smart appliances are useful for saving energy.
	PE2	I believe that smart appliances are effective in achieving energy saving.
	PE3	Using smart appliances increases my chances of achieving energy-saving goals.
	PE4	I can complete tasks faster with smart appliances.
	PE5	Using smart appliances can improve energy-saving performance.
EE	EE1	Learning how to use smart appliances is easy for me.
	EE2	I think smart appliances have easy-to-understand interfaces.
	EE3	I think smart appliances are very easy to use.
	EE4	I can become proficient in using smart appliances very quickly.
	EE5	The interaction process with smart appliances is clear and understandable.
SI	SI1	People I value think I should use smart appliances.
	SI2	People who influence me encourage me to use smart appliances.
	SI3	People I care about want me to use smart appliances.
	SI4	If smart appliances become popular, I’ll consider using them.
	SI5	Because many of my friends use smart appliances, I think I should use them too.
FC	FC1	I have the resources needed to use smart appliances.
	FC2	I know how to use smart appliances.
	FC3	Smart appliances are compatible with the devices and technologies I currently use.
	FC4	If my smart appliances have problems, I can ask for help.
	FC5	I can fix any problem with smart appliances.
UI	UI1	I’ll use smart appliances more often.
	UI2	I plan to use smart appliances in the future.
	UI3	Overall, I am very eager to use smart appliances.

*p < 0.05.

## Discussion

The current study aimed to construct a theoretical model for understanding the factors influencing consumers’ usage intention of smart home appliances. Our empirical analysis integrated trust, perceived source credibility, and personal innovativeness into the TTF and UTAUT models. This enabled us to overcome the limitations faced by previous studies and offer a more comprehensive understanding of consumers’ usage intentions in the context of smart home appliances. The most significant theoretical contribution of this study was that it created a new theoretical framework that combined two different theories of innovation adoption, namely, UTAUT and TTF, and incorporated factors such as trust, perceived source credibility, and personal innovativeness, thus offering a more comprehensive explanation for consumers’ usage intention of smart home appliances.

Our research findings showed that performance expectancy and effort expectancy had a significantly positive impact on usage intentions, which was in line with findings of previous research on UTAUT^10–12,26,31^. Performance expectancy, in particular, is considered to be the most important determinant of usage intention, while effort expectancy plays a relatively smaller role in determining consumers’ usage intention of smart home appliances. This indicates that consumers intend to use smart home appliances because these devices bring more convenience to their lives and help achieve the goals of energy conservation and environmental sustainability. Therefore, when developing the functionality of smart home appliances, practitioners should take into consideration consumers’ expectations toward it. This will help increase consumers’ intention to use smart home appliances. That being said, facilitating conditions had a significantly negative effect on usage intention, while social influence exhibited no influence^28^. This finding contradicted certain research reports, which identified social influence and facilitating conditions as predictors of the use of technology products^11,26,29^. Social influence did not significantly affect usage intention in this study. This finding aligns with Alwadain et al.^60^, who similarly reported that social influence had no significant impact on electric vehicle adoption within their integrated TTF-UTAUT framework. One possible explanation, consistent with Venkatesh et al.^11^, is that social influence tends to be more salient in mandatory adoption contexts and its effect diminishes in voluntary settings. Smart home appliance adoption is entirely voluntary, and the predominantly middle-aged composition of our sample (46.0% aged 40–49) may further attenuate social influence effects, as experienced consumers tend to rely more on personal assessments than on external social pressures^11^. Marikyan et al. also noted in their systematic review of smart home literature that social influence yields inconsistent effects across adoption studies, suggesting that its role is context-dependent and moderated by user characteristics^61^.

The negative effect of facilitating conditions on usage intention warrants careful consideration. This finding is not unprecedented; Chu et al. reported a similarly adverse effect of facilitating conditions on behavioral intention in the context of intelligent elevator adoption in Taiwan^62^. One plausible explanation is that when consumers perceive abundant existing resources and technical support, a sense of technological sufficiency may emerge, reducing their motivation to adopt additional smart home appliances. Consumers who believe their current setup adequately meets their needs may see less value in introducing new devices. An overemphasis on support infrastructure may also inadvertently signal product complexity, increasing consumers’ perceived burden rather than facilitating adoption. This interpretation should be treated with caution, as the present study did not include demographic variables as controls. Notably, Hossain et al. and Ly found positive effects of facilitating conditions on smart home adoption in developing countries (Bangladesh and Cambodia, respectively), suggesting that the direction of this relationship may vary across market maturity and consumer experience levels^63,64^. Future research should incorporate demographic covariates to better explain these divergent findings.

According to the research findings, all the hypotheses involving TTF were statistically supported. Both technology characteristics and task characteristics had a substantial influence on TTF, with the latter contributing more to TTF than the former. This corroborated the findings of previous research^9,27,32,41^. Meanwhile, TTF further determined users’ intention to use smart home appliances, which was consistent with previous research findings^9,15,41^. Hence, when promoting smart home services, practitioners should consider the extent to which the functionality of smart home appliances matches consumers’ task requirements, as well as their impact on environmental sustainability. In addition, they should also undertake market segmentation to analyze the need characteristics of different consumer groups and promote their use and usage behaviors.

TTF is a crucial antecedent of the four variables comprising the UTAUT model (performance expectancy, effort expectancy, social influence, and facilitating conditions), and it had a significant influence on facilitating conditions and effort expectancy. This finding resonates with Alwadain et al.^60^, who demonstrated that in their integrated TTF-UTAUT model for electric vehicle adoption, the direct effect of TTF on behavioral intention was non-significant while UTAUT constructs served as critical mediators. Our results extend this insight by showing that TTF significantly influences all four UTAUT constructs in the smart home context, establishing a comprehensive TTF-to-UTAUT mediation pathway. This demonstrates the robust association between TTF and the UTAUT model. Hence, good TTF is a key way to promote facilitating conditions and effort expectancy. If the functionality and services of smart home appliances obtained by consumers were to fall short of their needs, they would perceive them to be less useful. This may result in lower facilitating conditions and effort expectancy.

The research findings gave credence to the argument proposed by this study that perceived source credibility positively affects performance expectancy. This result is comparable to previous research findings^20^ and implies that highly credible information sources can boost consumers’ trust in the functionality and utility of a product^52^. Consumers who perceive an information source (such as brands, experts, or user reviews) to be credible are more likely to believe that a smart home appliance will produce the outcomes it claims to achieve and satisfy their needs and expectations. This reinforces their positive evaluation of the product, thus heightening their expectations regarding the performance of the appliance and influencing their purchase decisions and usage behaviors. The present study also argued that increasing trust has an influence on performance expectancy. This was supported by the empirical results, which were similar to previous research findings^22,42^. The results also imply that consumers who place their trust in the reliability and safety of smart home appliances are more likely to believe that these devices will function effectively and meet their needs as expected.

This study discovered that personal innovativeness significantly affects TTF and usage intention of smart home appliances. The results of hypothesis testing using the extended UTAUT model demonstrates that personal innovativeness emerged as the strongest direct predictor of consumers’ usage intention of smart home appliances (β = 0.518). While personal innovativeness also significantly influenced TTF (β = 0.085), this effect was modest compared with task characteristics (β = 0.642) and technology characteristics (β = 0.109). These results suggest that personal innovativeness primarily drives adoption through a direct motivational pathway rather than by enhancing perceived task-technology fit. This is consistent with Agarwal and Prasad’s^47^ conceptualization of personal innovativeness as a stable individual trait that predisposes technology-prone individuals toward early adoption, independent of contextual task-technology evaluations. Lastly, our findings also suggest that a wider range of explanations can be obtained for consumers’ use of smart home appliances by integrating the UTAUT and TTF models rather than by using either of the models alone. Therefore, future studies may combine these two theories to examine users’ adoption of other smart products or services. We believe that this would offer richer insights than individual research perspectives.

## Conclusions

This study investigated the factors influencing consumers’ intention to use smart home appliances in their family lives. To achieve this, two different theories of innovation adoption—namely, UTAUT and TTF—were integrated with three antecedents of consumer characteristics—personal innovativeness, trust, and perceived source credibility—to develop a comprehensive and unified model. Although several determinants of consumers’ use of smart home appliances had already been proposed by previous studies, the current study produced analytical findings that offered certain influence and contributions to existing literature. First, this study advances existing adoption theories by demonstrating that TTF functions as a cognitive appraisal mechanism that activates all four UTAUT constructs, establishing a TTF-to-UTAUT mediation pathway that offers a more nuanced explanation of the adoption process than either model provides independently. Second, the dual role of personal innovativeness, operating at both the appraisal stage (TTF) and the behavioral stage (usage intention), reveals the multi-level influence of individual difference variables in technology adoption. In the UTAUT model, performance expectancy and effort expectancy had a positive impact on usage intentions, facilitating conditions had a negative impact on usage intentions, and social influence had no such influence. Personal innovativeness had an influence on TTF and usage intentions toward smart home appliances.

Trust and perceived source credibility are two important antecedents of performance expectancy. Compared with a single model, our combined model exhibited stronger predictive power and contributed valuable insights for future studies. Third, this study emphasized the importance of considering personal innovativeness, trust, and perceived source credibility in the context of smart home appliances. The research findings provide practitioners with practical guidance and underscore the significance of promoting TTF, personal innovativeness, trust, and perceived source credibility to enhance consumers’ usage intention of smart home appliances. Finally, in the context of sustainable consumption, the use of smart homes has raised green living issues, and the construction of smart homes can improve the management of electricity, water, and gas consumption. The widespread adoption of smart home appliances will provide people with more convenient and personalized home services, which will help save energy and reduce carbon emissions. In addition, the findings of this study will help to understand how digital technology (especially smart home appliances) can promote sustainable consumption, providing policy makers. These insights will aid policymakers, developers, and stakeholders in the digital economy who seek to advance environmental sustainability through technological innovation.

## Limitations and recommendations for future research

Despite its theoretical and practical implications for clever home equipment usage intentions, this paper had several limitations. First, usage intentions toward smart home appliances was considered, however real behaviors may differ. Second, the absence of control variables presents another main problem. Demographic variables-which include age, profits, education, and prior experience with smart home equipment-may have a considerable effect on the connection between UTAUT/TTF constructs and utilization intentions. The sample’s age distribution, with 46.0% of respondents aged 40–49, may have influenced the observed relationships, particularly the non-significant effect of social influence and the negative effect of facilitating conditions. Similarly, other predictive factors aside from self-efficacy and task-technology may affect perceived ease of use and perceived usefulness in diverse contexts. For example, gender differences may affect the adoption of clever home appliances, and could be investigated by further research. Third, the generalizability of the research effects may be weak. Given that this survey was geographically localized to an area, it may not be reflective of other populations. This study was carried out using a survey technique, and future research could employ an experimental design technique. Fourth, respondents experience with smart home equipment or exposure to publicity of new technology may affect their perception and behavioral intentions. This can be controlled in future studies to better understand adoption of UTAUT/TTF as well as its boundary conditions. Fifth, we did not control whether the respondents have been homeowners, if they possessed clever appliances previously or if they are allowed to make decisions about utilizing smart appliances. Some may live at an apartment or other accommodation wherein they do not actually have a choice. Sixth, the measurement items for perceived source credibility focused on the credibility of energy-saving performance claims rather than the full spectrum of source credibility dimensions. Future research should develop more comprehensive measures that capture broader dimensions such as the expertise, trustworthiness, and attractiveness of information sources to strengthen construct validity. Additionally, the present study examined the effects of trust and perceived source credibility exclusively on performance expectancy. Future research could extend this investigation to explore whether these constructs also influence effort expectancy, social influence, or facilitating conditions, thereby providing a more comprehensive understanding of their roles within the UTAUT framework. Finally, the effect of additional variables (e.g., AI readiness, AI anxiety, and AI literacy) may to be tested in future research to understand their relationships among the focal variables.

## Data Availability

The data presented in this study are available on request from the corresponding author. The data are not publicly available due to privacy and confidentiality reasons.
